# Microbiology and postmortem interval: a systematic review

**DOI:** 10.1007/s12024-023-00733-z

**Published:** 2023-10-16

**Authors:** Bruna Moitas, Inês Morais Caldas, Benedita Sampaio-Maia

**Affiliations:** 1 Departamento de Ciências da Saúde Pública e Forenses e Educação Médica, Faculdade de Medicina, Universidade do Porto, Porto, Portugal; 2 Faculdade de Medicina Dentária da Universidade do Porto, Porto, Portugal; 3 CFE - Centre of Functional Ecology, University of Coimbra, Coimbra, Portugal; 4 1H-TOXRUN - One Health Toxicology Research Unit, University Institute of Health Sciences, CESPU, CRL, 4585-116 Gandra, Portugal; 5 i3S - Instituto de Investigação e Inovação Em Saúde, Universidade do Porto, Porto, Portugal; 6 INEB - Instituto Nacional de Engenharia Biomédica, Universidade do Porto, Porto, Portugal

**Keywords:** Postmortem interval, Microbiome, Microorganism, Bacterial DNA, Forensic microbiology

## Abstract

This systematic review aims to learn if and how it is possible to use the human microbiome to indicate the time elapsed after death. Articles were searched on the PubMed database using predefined data fields and keywords; reviews, systematic reviews, and meta-analyses were excluded. The final selection included 14 papers (out of 144). The results indicated that the microorganisms present in the cadaveric island succeed predictably over time, with markers between the stages of decomposition constituting a potential innovative tool for postmortem interval (PMI) estimation. The human microbiome has the potential to be used for PMI estimation and may present advantages as microbes are present in all seasons, in all habitats, including the most extreme ones, and because microbial communities respond predictably to environmental changes.

**Supplementary Information:**

The online version contains supplementary material available at 10.1007/s12024-023-00733-z.

## Introduction

Postmortem interval (PMI) is very important in forensic scenarios [1, 2], and traditional methods can offer comprehensive estimations of PMI [3, 4]. For example, entomology is based on the insects’ life cycle observation and characterization, which vary with external factors, being absent in cold seasons [5, 6]. Algor and rigor mortis are helpful mainly in the first 48 h after death [2]. The human microbiome has the potential to be used for estimating the PMI. After death, decomposition begins, with cellular autolysis leading to putrefaction caused by microbes [7]. As decomposition proceeds, anaerobic bacteria, mainly within the gut, produce gases resulting in bloating, followed by fluids’ purging and subsequent nutrients and microbes’ release into the underlying soil, altering its composition [8, 9]. Human microbiome analysis has advantages as microbes are present in all seasons and in all habitats, including the most extreme ones [1, 2, 10, 11]. Moreover, because microbial communities respond predictably to environmental changes, it is possible to establish a succession of the bacterial community over time and, therefore, estimate PMI [12, 13]. The analysis of the necrobiome is done by studying the microorganisms colonizing internal organs [14], body fluids [15], orifices after death (thanatomicrobiome), and those present on the cadaver surface (epinecrotic microbiota communities) [4, 16].

This systematic review aims to understand the microbial succession changes during decomposition and how the human microbiome can be used to indicate PMI.

## Material and methods

This review followed the PRISMA (Preferred Reporting Items for Systematic Reviews and Meta-Analyses) protocol [17] and was registered on the PROSPERO (International Prospective Register of Systematic Reviews) (registration number CRD42022292235). The articles were selected from PubMed database (October to December 2021) with the query: (“microbiology” or “microbiome” or “*Streptococcus*” or “microorganism” or “microbes” or “microflora” or “microbial” or “bacteria” or “fungi” or “yeast” or “Candida”) AND (“post-mortem interval” or “postmortem interval”). This query is intended to respond to the following PICO (Population, Intervention, Comparison and Outcome) question [18]: “In human cadavers, how can microbiology assist forensic science practice through the analysis of microbial ecosystem changes throughout corpse decomposition as a tool for postmortem interval estimation?”. Reviews, systematic reviews, and meta-analyses were excluded. Articles were selected progressively, reading the title, the abstract, and, finally, the full text. Each article eligibility assessment was made independently by all authors, and disagreements were resolved by consensus. Data were extracted from each study and organized into a table, including title, authors, year of publication, population, the type of study, the main objective, the intervention, and the outcome. The risk of bias was analyzed using the Joanna Briggs Institute protocol [19]. Articles were classified with “no,” “yes,” or “unclear” for each question present in the protocol. For each “yes,” a point was given, and articles scoring six or more were selected.

## Results

**Fig. 1:**
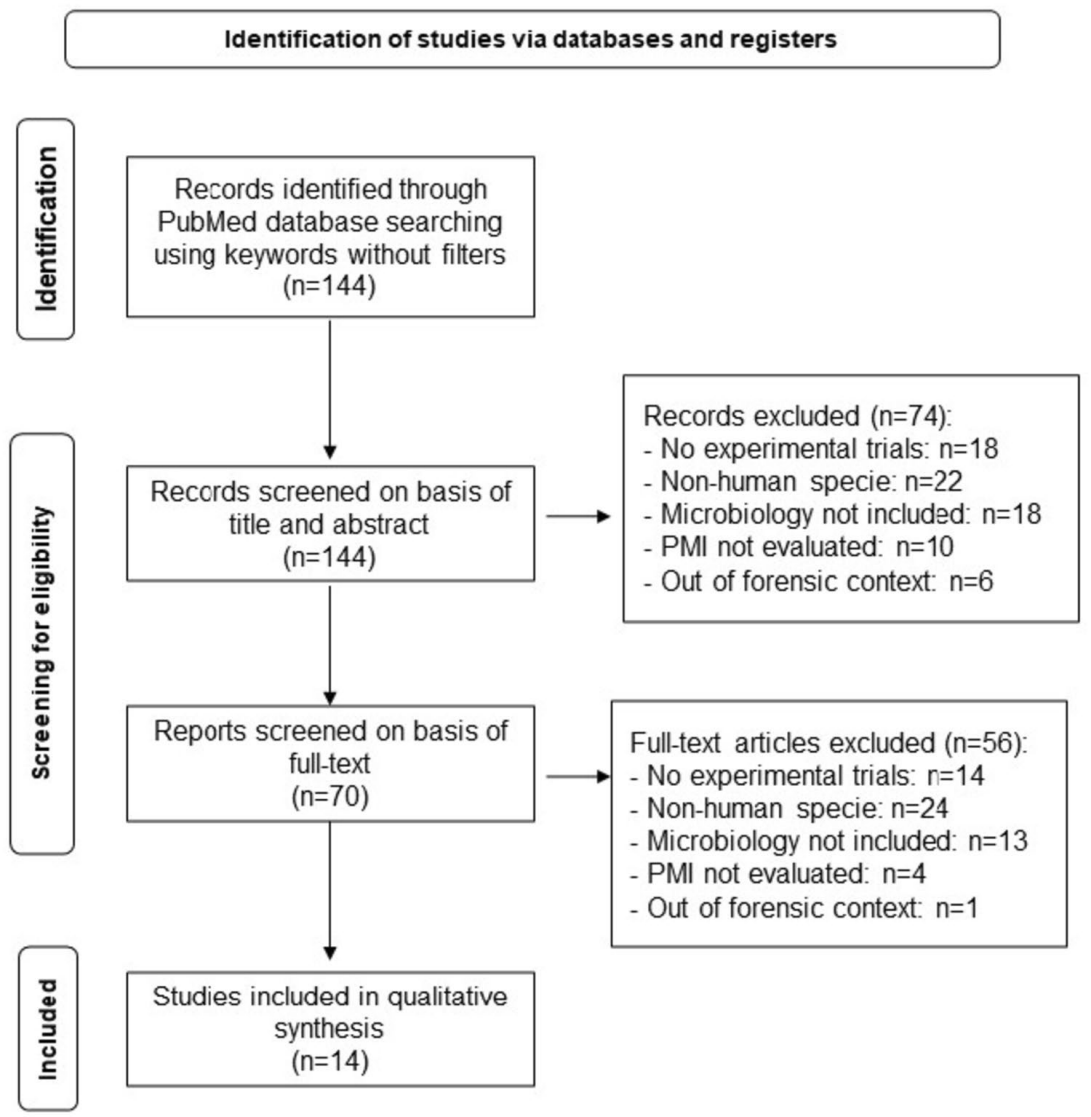
Systematic review flowchart leading to selection of articles

From the 144 articles obtained, 14 were included for analysis and data extraction (Fig. 1). These were experimental studies, published in English, and presented a low risk of bias analysis (Supplementary Table 1). All articles assessed the changes in microbial communities throughout the cadaveric decomposition process and their use for estimating PMI (Table 1). Twelve articles explored the bacterial community [7, 12–14, 20–27]. From those, the bacterial community was evaluated by 16S rRNA gene amplicon high-throughput sequencing in 11 articles [7, 12–14, 20–27]; by qPCR targeting *Bacteroides*, *Lactobacillus*, and *Bifidobacterium* in one paper [23]; by 16S rRNA gene high-throughput sequencing combined with metagenomic and metatranscriptomic sequencing and culture in selective and rich media in one paper [27]; and by 16S rRNA and 18S rRNA amplicon high-throughput sequencing to explore also the eukaryotic communities in another [26]. One paper explored the eukaryotic fungi ecosystem through culture in selective media and photography to macroscopically monitor the area of fungal coverage [28], and another paper evaluated the microbial neoformation of volatiles using headspace gas chromatography with flame ionization identification method (HS-GC-FID) [29]. Moreover, different studies focused on the microbial ecology of other anatomic areas: head external orifices (hard palate, mouth, nose, ears, and eyes) [13, 21, 24, 25, 27], gut [12, 23], internal organs and blood [14, 29], skin [28], and bones [22, 26]. Also, the interactions between corpse and soil microbiomes were accessed [7, 20, 22, 26].

**Table 1:** Summarisation of the information obtained from the articles under analysis

**Article**	**Participants**	**Place the study was performed**	**Main goal**	**Microbial group evaluated**	**Method of microbial assessment**	**Major findings**	**PMI determination**
Adserias-Garriga et al. (2017) [20]	3 cadavers under environmental conditions followed up to 12 days	University of Tennessee Anthropology Research Facilities in Knoxville, Tennessee	Evaluate the temporal effects of human cadaver decomposition on the soil microbiome to estimate the PMI	Bacteria in soil (surrounding the cadavers)	16S rRNA gene (V4 region) amplicon high-throughput sequencing	Bacterial communities present in the soil surrounding the cadavers changed while the decomposition of the bodies was taking place. Bacteria from the decomposing body were transferred from the cadaver to the soil and, therefore, the abundance of indigenous bacterial soil communities (mostly Pseudomonadota, Acidobacteriota, Bacteroidota), present at first stages of decomposition, progressively decreased. Days 6 and 7, coincident with an active, advance decay, or bloat stage, represented a breakpoint consisting of a sudden increase of Bacillota and Actinomycetota that was concomitant to a decrease of Pseudomonadota. Final stages of decomposition (advance decay and dry remains) are characterized by a high abundance of Clostridiales (Bacillota) in the soil underneath the mouth and abdominal areas, whereas around the feet area Pseudomonadota is the most abundant phylum, followed by Bacillota. The growth curve of Bacillota in the soil under human remains can be an interesting tool to estimate time since death during Tennessee summer conditions	YES
Adserias-Garriga et al. (2017) [21]	3 cadavers under environmental conditions followed up to 12 days	University of Tennessee Anthropology Research Facilities in Knoxville, Tennessee	Evaluate the temporal effects of human cadaver decomposition on the oral microbiome to estimate the PMI	Bacteria in oral cavity	16S rRNA gene (V4 region) amplicon high-throughput sequencing	The oral microbiome suffers successional changes throughout decay. The oral microbiome was characterized i) in the fresh stage by indigenous oral commensals, such as Lactobacillaceae, Staphylococcaceae, Gemellaciae, Carnobacteriaceae, Aerococcaceae, Veillonellaceae, Streptococcaceae, Campylobacteraceae, Micrococcaceae, Bifidobacteriaceae, Actinomycetaceae, and Corynebacteriaceae; ii) in the bloat stage by both oral (Peptostreptococcaceae and Bacteroidaceae) and gut (Enterococcaceae, Clostridiales); iii) in advance decay mainly by soil microbiota (Gamma-Proteobacteria, Pseudomonadaceae, Alcaligenaceae, and Planococcaceae); and iv) in dry remains by Bacilli and Clostridia. As the decomposition process advance, bacterial communities change according to the newly set environmental conditions, where oxygen availability strongly determines the temporal ecosystem dynamics	YES
Ashe et al. (2021) [27]	3 cadavers	Forensic Osteology Research Station (FOREST) at Western Carolina University	Evaluate the temporal effects of human cadaver decomposition on the human oral microbiome (hard palate) to estimate the PMI	Bacteria in hard palate	16S rRNA sequencing combined with metagenomic (MetaG) and metatranscriptomic sequencing (MetaT) and culture in selective and rich media	Pseudomonadota, Bacillota, and Actinomycetota were the dominant phyla, but their distributions were insufficient in separating samples based on decomposition stage or time or by donor. Better resolution was observed at the level of genus, where a distinction could often be made between the community from fresh vs. later sampling times. Taxa from the normal human oral microbiome before death (e.g., *Lactobacillus*, *Streptococcus*, *Rothia*, and *Candida*) was replaced as decomposition progressed by *Lysinibacillus*, *Vagococcus*, *Ignatzschineria*, and *Yarrowia*. MetaG and MetaT data also revealed many novel insects as likely visitors to the donors in this study, opening the door to investigating them as potential vectors of microorganisms during decomposition. *Notwithstanding, a* finer resolution is required for PMI estimation because there was no apparent relationship between the length of the PMI and the bacterial community present at a given time	NO
Ceciliason et al. (2021) [29]	412 autopsied corpses with PMI between 0 to 106 days	Departments of Forensic Medicine in Uppsala and Gothenburg, Sweden	Determine if microbial neoformation of volatiles could be used as a tool to improve the precision of PMI estimation	Microbial neoformation detected in vein blood (collected at autopsy)	Headspace gas chromatography with the flame ionisation detection method (HS-GC-FID)	The most common microbial-derived volatiles found were acetaldehyde (83% of the cases), followed by ethanol (37%), N-propanol (21%), and 1-butanol (4%). The results indicated no or weak linear relationship between the PMI and the detected volatiles. Cases positive for N-propanol and/or 1-butanol had, however, a higher degree of decomposition. Among the 4 volatiles evaluated, N-propanol and 1-butanol are the most promising indicators of the decomposition rate within the early decomposition to bloating stages, while ethanol levels may be affected to a varying extent by antemortem intake of alcoholic beverages	NO
Damann et al. (2015) [22]	12 cadavers under environmental conditions followed up to 1446 days	University of Tennessee Anthropology Research facility	Evaluate the temporal effects of human cadaver decomposition on skeletonized remains to estimate the PMI	Bacteria in partially and fully skeletonized and dry remains (human ribs) and in soil (from 1 km away)	16S rRNA gene (V3 region) amplicon high-throughput sequencing	Taxonomic succession based on relative abundance of specific bacterial phyla may prove useful for estimating a PMI of skeletonized remains. For example, the partially skeletonized remains (PMI:27–284 days) presented the highest proportion of Bacillota; the fully skeletonized remains (PMI:292–369 days) presented highest relative abundance of Bacteroidota; and the dry remains (PMI:554–1692 days) hosted the largest proportion of Actinomycetota. Therefore, the interstage taxonomic succession of Bacillota, Bacteroidota, Actinomycetota, and Acidobacteriota from decaying bone suggested an underlying continuous transition in community composition, where partially skeletonized remains maintained a presence of bacteria associated with the human gut, and bacterial composition of dry skeletal remains maintained a community profile similar to soil communities. Community membership (unweighted) may be better for estimating PMI from skeletonized remains than community structure (weighted)	YES
DeBruyn and Hauther (2017) [12]	4 cadavers under environmental conditions followed throughout decay	Anthropology Research Facility at University of Tennessee, Knoxville	Evaluate the temporal effects of human cadaver decomposition on the gut microbiome to estimate the PMI	Bacteria in gut	16S rRNA gene (V4 region) amplicon high-throughput sequencing	Over time, the taxon richness of the bacterial communities increased, while the diversity significantly decreased. “Early” microbial communities had high diversity (major phyla were Bacillota and Bacteroidota) whereas the “late” microbial communities had a higher richness but lower diversity (enriched by Clostridiales and Gamma-Proteobacteria). The bacterial communities had a shift in the middle of the bloat phase of decomposition, between days 4 and 7 (this shift may correspond to a change in the physical/chemical environment and/or biological competition). OTUs belonging to Bacteroidales (*Bacteroides*, *Parabacteroides*) significantly declined while Clostridiales (*Clostridium*, *Anaerosphaera*) and the fly-associated Gamma-Proteobacteria *Ignatzschineria* and *Wohlfahrtiimonas* increased throughout decay. *Clostridium* was the strongest positive predictor of PMI	YES
Deel et al. (2021) [26]	6 cadavers under environmental conditions followed up to 9 months	Southeast Texas Applied Forensic Science Facility	Evaluate the temporal effects of human cadaver decomposition on the human bone microbiome to estimate the PMI	Bacterial and eukaryotic microorganisms in human ribs	16S rRNA gene (V4 region) and 18S rRNA amplicon high-throughput sequencing	The core bone decomposer microbiome increased as decomposition progressed, was different between seasons (spring and summer), and was dominated by taxa of the bacterium phylum Pseudomonadota, Bacillota, Actinomycetota, and Bacteroidota and of eukaryotic phylum (or subdivisions) Ascomycota, Nematoda, Basidiomycota, Apicomplexa, and Ochrophyta. This bone decomposer microbiome likely originates from the surrounding decomposition environment, including skin of the cadaver and soil. Modelling results indicate that 16S rRNA data are more accurate in estimating PMI than the 18S rRNA data. *Beta-diversity,* Phyllobacteriaceae*, and Devosia* appear to be particularly useful for predicting PMI of bone remains. This work showed that the 16S rRNA gene analysis either in spring or summer may have the potential to generate probative PMI estimates	YES
Di Piazza et al. (2018) [28]	2 cadavers preserved in controlled condition followed for 6 weeks	Hospital Mortuary	Evaluate the temporal effects of human cadaver decomposition on the total body fungal colonization to estimate the PMI	Fungi in the corpse	Culture in selective media and photograph to monitor the area of fungal coverage	Mycological diversity in colonization patterns is caused by the interaction of initial inoculum, initial conditions of the corpse and environmental parameters in the day close the death. Fungi colony maturation (colour and conidiogenesis) is essential to estimate PMI. The different fungal species colonizing the corpse may aid in geolocation determination and in estimating the environmental climatic parameters where the body laid between the perimortem and postmortem periods	YES
Ceciliason et al. (2021) [29]; Hauther et al. (2015) [23]	12 cadavers under environmental conditions, 6 followed up to 20 days	Anthropological Research Facility in Knoxville, Tennessee	Evaluate the temporal effects of human cadaver decomposition on the gut microbiome to estimate the PMI	*Bacteroides*, *Lactobacillus* and *Bifidobacterium* in the gut	Quantitative PCR using targeted primers	*Bacteroides* and *Lactobacillus* relative abundances declined exponentially with increasing PMI. Contrarily, PMI did not seem to significantly alter Bifidobacterium abundances. Repeated sampling affected *Bifidobacterium* abundances, likely due to the introduction of oxygen to the body cavity, but not *Bacteroides* and *Lactobacillus populations*. No significant effects of sex, weight, or cause of death were observed	YES
Javan et al. (2016) [13]	27 cadavers from criminal cases with PMI between 3,5 and 240 h	Alabama and Florida morgues	Evaluate the temporal effects of human cadaver decomposition on the thanatomicrobiome to estimate the PMI	Bacteria in blood, brain, oral cavity, heart, liver, and spleen	16S rRNA gene (V4 region) amplicon high-throughput sequencing	The thanatomicrobiome signatures showed time-, organ-, and sex-dependent changes useful for PMI estimation. Family and genus level analyses explained approximately 21% of variance in models correlating PMI whereas species level analysis explained 65%. Several genera, such as *Clostridium* and *Prevotella*, include members potentially predictive of different periods of decomposition (eg., *Clostridium novyi* more abundant at the latest PMI whereas an unknown *Clostridium* species was more abundant earlier in decomposition). Bacillota (which includes *Clostridium*) appear to constitute a stable biomarker across thanatomicrobiome communities. Bacterial genera were similar among different organs within each sex, but were dissimilar between females and males, with the exception of oral cavity, where similar genera were seen for both sexes	YES
Johnson et al. (2016) [24]	21 cadavers under environmental conditions, followed throughout decay	Anthropological Research Facility at the University of Tennessee	Evaluate nasal and ear canals microbiota during decay to establish an algorithm for predicting the PMI	Bacteria in nasal and ear canals	16S rRNA gene (V3-V4 region) amplicon high-throughput sequencing	The ear microbiome diversity tends to be negatively correlated with the decay process. Complete data set, rather than a curated list of indicator species, and genus and family was preferred to improve the PMI´s estimation. Predictive model exhibited greater accuracy and was useful over a long period of decomposition time. Skin microbiota is a promising tool in forensic death investigations	YES
Lutz et al. (2020) [14, 30]	40 autopsied corpses (14 females and 26 males)	Department of Health, Experimental and Forensic Medicine at the University of Pavia, Italy	Evaluate the effects of human cadaver decomposition on the internal organs microbiome to estimate the PMI	Bacteria in brain, heart, liver, spleen, prostate, and uterus (collected at autopsy)	16S rRNA gene (V4 region) amplicon high-throughput sequencing	With increasing PMI, was observed a significant increase in relative abundance of Burkholderiales in the heart and Clostridiales in the brain, liver, and spleen. Also, PMI was associated with microbial alpha and beta diversity. Other cadaver-specific traits (sex, BMI, and cause of death) were also associated with microbial alpha and beta diversity and different bacterial taxa. Nulligravid uterus and prostate revealed to be the last organs to putrefy during decomposition	YES
Singh et al. (2017) [7]	11 cadavers under environmental conditions followed up to 732 days	Forensic Anthropology Research Facility of Texas University, San Marcos, Texas, USA	Evaluate the temporal and spacial effects of human cadaver decomposition on soil bacteria to estimate the PMI	Bacteria in soil (under the cadavers)	16S rRNA gene (V1-V3 region) amplicon high-throughput sequencing	In the presence of a cadaver, bacterial community composition at 0 m deep was significantly different from 1 and 5 m: the relative abundance of Bacteroidota and Bacillota was greater, while the relative abundance of Acidobacteriaota, Chloroflexota, Gemmatimonadetes, and Verrucomicrobiota was lower. Also, the relative abundance of Actinomycetota increased significantly with cumulative precipitation. Gamma-Proteobacteria, but not Bacilli, positively associated with cadaver starting weight. Decomposition alters soil bacterial communities’ structure and microbial function directly under cadavers, up to 2 years. Predictive models of a PMI with such data may be accurate or precise if they include accumulated precipitation and the starting weight of a decadent	YES
Zhang et al. (2019) [25]	188 cases on dataset	Samples were obtained at Wayne County Medical Examiner’s Office (Detroit, Michigan)	Evaluate machine learning methods performance for PMI estimation using microbial community analyses, focusing on the number of samples and the anatomical areas	Bacteria in auditory canal, eyes, nose, mouth, and rectum	16S rRNA gene (V4 region) amplicon high-throughput sequencing	When predicting the PMI, the highest accuracy (77,5%) was achieved when all five anatomic areas were used. *Veillonella dispar* sp. and *Proteus sp*. Counts were higher in cases with a PMI greater than 73 h while Moraxellaceae had a higher count in cases with PMI 49–72 h, *Streptococcus* sp. count was higher within a 48 h PMI. As decomposition progresses, postmortem microbial community richness and diversity significantly decreases after a PMI of 48 h or greater, suggesting that community information may be more useful than individual taxa. Analysis of postmortem microbiota from more than three anatomic areas appears to yield limited returns on accuracy, with the eyes and rectum providing the most useful information correlating with circumstances of death in most cases for this dataset	YES

PMI was measured in hours, days, months, or years or as accumulated degree days (ADD). ADD is the cumulative total of daily average temperatures. Linking the decomposition stages or insect development to ADD allows temperature variations to be considered when estimating PMI [31]. Cumulative degree hours (CDH), a refinement of the ADD calculation process for cadavers decomposing over a short period, represent an average value for each 12-h interval. Similarly to ADD calculation, temperatures below 0 °C are counted as a zero, and negative values are not used [32, 33]. The Total Body Score method (TBS), a scale that distinguishes decomposition different stages, allowing to assign points to specific categories and eventually to score overall decomposition [34, 35], was also used [29].

For clarity, the description of the individual studies will be organized by the anatomical areas, when possible. For detailed and organized data, please refer to Table 1. Adserias-Garriga et al. [21] took oral swab samples from three cadavers at different putrefaction stages; all showed similar successional changes, despite having other oral conditions. Bacillota (previously named Firmicutes) and Actinomycetota (previously called Actinobacteria) were the most predominant phyla from day 1 to 5, coinciding with the fresh stage. Their relative abundances decreased from day 1 to day 5–6, whereas Tenericutes (gastro-intestinal microbiota) appeared at the bloat stage (days 5 and 7). The oral microbiome was characterized: i) in the fresh stage by indigenous oral commensals; ii) in the bloat stage by both oral (Peptostreptococcaceae, Bacteroidaceae) and gut (Enterococcaceae, and Clostridiales) microbiota; iii) in advance decay, mainly by soil microbiota; and iv) in dry remains, by bacilli and clostridia. The authors suggest oxygen availability is a determinant factor for bacterial ecological changes.

Ashe et al. [27] sampled three cadavers’ hard palate, and samples were analyzed by combining 16S rRNA sequencing with whole metagenomic (MetaG) and metatranscriptomic (MetaT) sequencing and cultured in selective and rich media. The analysis by different sequencing techniques allowed a more complete view of the microorganisms present at the various decomposition. Concerning the 16S taxonomic distributions, 25 to 70% were Bacillota in the fresh decomposition phase (ADD values up to 50); Pseudomonadota (previously named Proteobacteria) were found in most samples, and Actinomycetota were more numerous in 2 out of 7 samples; in one sample, Actinomycetota consisted of 75% due to the genus *Rothia*. Middle to later decomposition stages (> ADD 168) reveal that Bacillota dominated (> 75% sequences) or co-dominated (~ 50% sequences) in 9 of 13 samples; Pseudomonadota dominated or co-dominated in 5 of 13 samples, and in one sample (291 ADD), Actinomycetota registered more than 50% of sequences. *Pseudomonas* spp. was most common in later samples. Regarding MetaG Taxonomic distributions, *Streptophyta* (from Plantae kingdom) were common (11 of 17 samples), Bacillota were found in earlier samples, and Pseudomonadota were higher in later samples. On the taxonomic distributions of MetaT, bacterial data showed that Bacillota were common for the early and middle samples, Pseudomonadota were generally lower (except for the later stages samples); Actinomycetota were found in lower numbers in both early and later samples. Cultures included 46 bacterial unique species from 69 isolates. Species from the Pseudomonadota accounted for the isolates’ largest proportion (43.5%). Bacillota (32.6%), Actinomycetota (19.6%), and Bacteroidota (previously named Bacteroidetes) (4.3%) accounted for the rest. In total, 4 phyla were identified by culture, 7 by 16S sequencing, 66 by MetaG sequencing, and 51 by MetaT sequencing, more than half bacterial. Bacillota, Pseudomonadota, and Actinomycetota were the most abundant. Still, their distributions did not distinguish samples based on decomposition stage or time or by donor. However, Bacillota were more common in the early and Pseudomonadota in the later stages. Better resolution was observed at the genus level, where a distinction could often be made between the community from fresh (ADD < 50) vs. later (ADD > 168) sampling times. Taxa from the standard living human oral microbiome (e.g., *Lactobacillus*, *Streptococcus*, *Rothia*, and *Candida*) were replaced as decomposition progressed by *Lysinibacillus*, *Vagococcus*, *Ignatzschineria*, and *Yarrowia*. Still, a relationship between PMI and the bacterial community present during sampling was not established since only differentiation between fresh and late states was considered.

Another study [25] aimed to evaluate the machine learning methods’ performance on microbial community analysis obtained from 188 cases and postmortem samples from different anatomical areas (ears, eyes, nose, mouth, and rectum). Machine learning methods are tools that implement neural network and random forest models to perform regression and feature selection tasks on microbiome data; these methods allow the assessment of large multi-dimensional datasets that otherwise would be difficult to analyze and interpret. In the study mentioned above, for PMI prediction (i.e., their abilities to predict PMI), the highest accuracy (77.5%) was achieved when all anatomic areas were used. The most relevant microbial taxa identified were *Veillonella dispar* and *Proteus* sp. (PMI > 73 h). In comparison, Moraxellaceae had a higher count in cases with 49–72 h estimated PMI, and *Streptococcus* sp. within 48 h PMI. Thus, microbial signatures change between the time frames less than 2 days postmortem and more than 2 days postmortem, with an increasing number of unique taxa associated with communities in the first 2 days. As decomposition progresses, the postmortem richness (the total number of species in a community) and diversity (the number of species and their abundance in a community) of the microbial community decrease significantly (PMI ≥ 48 h).

Johnson et al. [24] also used a machine-learning approach, with 114 microbial samples collected from 21 cadavers’ ears and nasal cavities. The best methods and PMI indicators were evaluated to establish an algorithm to predict PMI from microbial samples, using three computational methods: *F*-value (feature selection considers the coefficient resulting from fitting a single feature with the target using a linear model), a tree-based approach (ranks features on their tendency to occupy essential positions in decision trees built on the same dataset), and mutual information (scores each taxon on the amount of information it has in common with the dependent variable). The results showed that the entire data set analysis works best, rather than any specific taxon or even small group of taxa; some taxa were identified as powerful indicators of PMI: phyla Actinomycetota and Armatimonadota and the classes Thermoleophilia and Erysipelotrichi were marked as top results by all three methods; the orders Myxococcales and Erysipelotrichales, the families Staphylococcaceae, Planococcaceae, and Enterococcaceae, and genera *Staphylococcus* and *Vagococcus* were identified as essential features by two of three methods. A correlation between microbial diversity and ADD was proven; the ear microbiome diversity was negatively correlated with ADD, and the nasal microbiome was positively correlated but less pronounced. Additionally, analyzing taxon at multiple levels simultaneously and combining samples from different body sites improved the model’s accuracy.

In the Hauther et al. study [23], *Bacteroides*, *Lactobacillus*, and *Bifidobacterium* were quantified in the gut by qPCR using targeted primers in 12 cadavers (including 6 controls, sampled only once). For 20 days, corresponding to 600 CHD, *Bacteroides* and *Lactobacillus* relative abundances declined exponentially with increasing PMI. Contrarily, PMI did not significantly alter *Bifidobacterium* abundance. Repeated sampling affected *Bifidobacterium* abundance, likely due to oxygen introduction but not *Bacteroides* and *Lactobacillus* populations. This study shows that the gut *Bacteroides* and *Lactobacillus* populations may be used as a PMI indicator at these intervals, as both populations decline with PMI.

The DeBruyn and Hauther study [12] also investigated the postmortem changes in the gut microbiome of 4 cadavers for 30 days after death. The bacterial communities’ taxon richness increased with time while the diversity decreased. An “early” and “late” microbial structure was defined, with changes occurring at the bloat stage (4 to 7 days). In the “early” microbial communities, the diversity was high, with the predominant phyla being those characteristics of the human gut microbiome: Bacteroidota and Bacillota; the “late” microbial communities had a higher richness but had lower diversity since the relative abundance of Bacteroidota declines. Although Bacillota still dominated, these communities were significantly enriched with microorganisms belonging to Clostridiales order and the fly-associated Gamma-Proteobacteria. *Bacteroides* and *Parabacteroides* declined over time and were significantly inversely correlated to PMI, and *Clostridium* was a PMI strong positive predictor.

In the Lutz et al. study [14], postmortem microbial DNA was extracted from several organs (brain, heart, liver, spleen, prostate, and uterus) of 40 Italian cadavers to investigate variation and microbial associations among different body organs in human cadavers to predict PMI. The different organs’ bacterial communities’ analysis showed significant differences in the relative abundances of multiple taxa, as the non-reproductive organs were dominated by bacterial orders MLE1-12, Saprospirales and Burkholderiales*.* In contrast, reproductive organs were dominated by Clostridiales and Lactobacillalaes and showed a marked decrease in relative abundance of MLE1-12. Several significant relationships were identified regarding the association between PMI and bacterial relative abundances. Within the heart, taxa belonging to the order Burkholderiales exhibited a significant increase in relative abundance with increasing PMI. In all organs, except for the uterus, taxa belonging to the order Clostridiales demonstrated an increase in relative abundance with increasing PMI (only significant for the brain, liver, and spleen). In the brain, heart, liver, and spleen, taxa of the order MLE1-12 showed a slight decrease in relative abundance with increasing PMI but not significant.

Javan and colleagues [13] analyzed the 20 predominant bacterial genera relative abundances in 66 samples of different human body organs (brain, mouth cavity, heart, liver, and spleen) and blood collected from 27 human corpses, with a PMI between 3.5 and 240 h. The thanatomicrobiome signatures showed time-, organ-, and sex-dependent changes useful for PMI estimation. Family and genus-level analyses explained approximately 21% of the variance in models correlating PMI, while species-level study explained 65%. Several genera, such as *Clostridium* and *Prevotella*, could predict different decomposition periods. The phylum Bacillota was identified as a possible biomarker in the thanatomicrobial communities from other body locations. Although the phylum was not a strong PMI predictor, Bacillota genera such as *Clostridium*, *Bacillus*, *Peptoniphilus*, *Blautia*, and *Lactobacillus* exhibit temporal changes. Bacterial genera were similar among different organs within each sex but dissimilar between females and males, except in the oral cavity. Females had a higher relative abundance of *Pseudomonas* and *Clostridiales*, while males had *Clostridium*, *Clostridiales*, *Streptococcus*, and *Rothia*.

Ceciliason et al. [29] analyzed blood collected from 412 corpses to assess the presence of ethanol, N-propanol, 1-butanol, and acetaldehyde. The volatiles were analyzed by HS-GC-FID. The decomposition degree was evaluated in three anatomical regions based on external visual signs. The obtained dataset was divided into non-decomposition and external decomposition cases, and PMI ranged from 0 to 6 days in the first group and 0 to 106 days in the second. The most common microbial-derived volatiles found were acetaldehyde (83%), followed by ethanol (37%), N-propanol (21%), and 1-butanol (4%). The results indicated no or weak linear relationship between PMI and detected volatiles, probably because this study accessed an indirect measure of the microbial community.

Fungal colonies were identified on the cadaver surface (skin), and their macromorphological variation was used to assess their potential as a PMI marker [28]. Photographs to monitor the fungal coverage area were made, and samples were collected for culture and identification of fungi. Initially, fungal colonies appeared on the face, right arm, and both ankles; during the following weeks, the colonies spread to the arms, chest, abdomen, autopsy suture, groin, and legs. In the more colonized areas, the corpse tissues appear to have a higher level of dehydration compared with the non-colonized areas. Also, a quite homogeneous and gradual growth on the face was observed and accompanied by a chromatic variation of each colonized area in relation to the time development. In another case, fungal growth was limited to the oral cavity, and the colonies observed were *Penicillium expansum* and *Cladosporium cladosporioides*, with colonization beginning on the palate 2 days after death. The colonization pattern diversity is caused by the interaction of the initial inoculum, initial conditions of the corpse, and the environmental parameters in the perimortem period; therefore, the corpse mycobiota characterization may also reveal possible changes in the corpse’s location.

Damann’s study [22] aimed to evaluate the bacterial communities’ potential as a method for estimating PMI for long periods, assess the differences in the microbiome between partially skeletonized remains (PMI = 27–284 d (days)), skeletonized samples (PMI = 292–369 d), and dry remains (PMI = 554–1692 d). In parallel, soil samples were collected for comparison to the ribs microbiome. Partially skeletonized remain samples, followed by the fully skeletonized, presented the lowest taxonomic diversity level. Pseudomonadota was the most abundant in all sample groups. Alpha-Proteobacteria increased relative abundance for each successive bone decay stage, while Gamma-Proteobacteria decreased. After the Pseudomonadota, members of the Bacillota and Bacteroidota were the most abundant, mostly present in the gut microbiota. Actinomycetota, prevalent in gut and soil microbiomes, and Acidobacteriota, present exclusively in the environment, were more prevalent in dry than partially/fully skeletonized remains. The partially skeletonized remains (PMI:27–284 days) presented the highest proportion of Bacillota; the fully skeletonized remains (PMI:292–369 days) showed the highest relative abundance of Bacteroidota; and the dry remains (PMI:554–1692 days) hosted the most significant proportion of Actinomycetota. The interstage taxonomic succession from decaying bone suggested an underlying continuous transition in community composition, partially skeletonized remains maintained a presence of bacteria associated with the human gut, and the dry skeletal remains bacterial composition kept a community profile like soil communities. This work also showed that community membership (unweighted) may be better for estimating PMI from skeletonized remains than community structure (weighted). Thus, bacterial community members can be a temporal reference for estimating skeletonized remains PMI.

Deel et al. [26] placed six unclothed corpses outdoors, three in the spring and three in the summer. A rib was collected from each body for eight time points, 3 weeks apart. A linear mixed-effects model was used to support the microorganisms invading the bone diversity increased throughout decomposition, with significant differences over time for both prokaryotic and eukaryotic organisms. The core bone decomposer microbiome: a) increased as decomposition progressed, b) was different between seasons, and c) dominated by taxa of the bacterium phylum Pseudomonadota, Bacillota, Actinomycetota, and Bacteroidota, and eukaryotic phylum (or subdivisions) Ascomycota, Nematoda, Basidiomycota, Apicomplexa, and Ochrophyta. Modeling results indicated that 16S rRNA data were more accurate in estimating PMI than the 18S rRNA data. Bacterial community composition became increasingly different from the initial community as decomposition progressed, with the community composition change rate decreasing over ADD, indicating a repeatable succession of invading microbes. Effect size calculations showed that ADD had the highest effect on beta diversity in nearly every case (particularly for the 16S rRNA data). Two taxa, *Phyllobacterium* and *Devosia*, increased in prevalence at higher ADD, providing information about the decomposed bone ecology over time. They concluded that the 16S rRNA gene analysis, in both seasons, may generate probative PMI estimates, with an error of ± 34 days, being more accurate than the current method for skeletonized remains. Beta-diversity, Phyllobacteriaceae, and *Devosia* (belonging to Alpha-Proteobacteria, primarily present in the soil) were considered particularly useful for predicting PMI from bone remains.

Adserias-Garriga et al. [20] showed that the bacterial communities in the soil surrounding the cadavers changed during decomposition. Bacteria were transferred from the cadaver to the soil; therefore, the abundance of indigenous bacterial soil communities (mostly Pseudomonadota, Acidobacteriota, and Bacteroidota) at the decomposition first stages progressively decreased. Days 6 and 7, coincident with an active, advanced decay, or bloat stage, represented a breakpoint, with a Bacillota and Actinomycetota sudden increase, concomitant to a Pseudomonadota decrease. The final stages of decomposition were characterized by a high abundance of Clostridiales (Bacillota) in the soil underneath the mouth and abdominal areas. In contrast, around the feet area, Pseudomonadota was shown to be the most abundant phylum, followed by Bacillota.

The Singh et al. study Campo [7] assessed human body decomposition’s temporal and spatial effects on soil bacterial communities. The bacterial community composition under the corpse (0 m) was significantly different from the one at 1 m and 5 m deep (which were similar). An increase in the relative abundance of classes Actinomycetota, Gamma-Proteobacteria, and bacilli at 0 m samples compared to 1 and 5 m samples was verified; similarly, the relative abundance of Bacteroidota and Bacillota was greater, while the relative abundance of Acidobacteriota, Chloroflexota, Gemmatimonadota, and Verrucomicrobiota was lower when compared to the 1 and 5 m samples. Also, this study showed that decomposition altered soil bacterial communities’ structure and microbial function directly under corpses for up to 2 years. Moreover, the relative abundance of Actinomycetota increased significantly with cumulative precipitation and Gamma-Proteobacteria, but not bacilli, positively associated with cadaver starting weight.

## Discussion

**Fig. 2:**
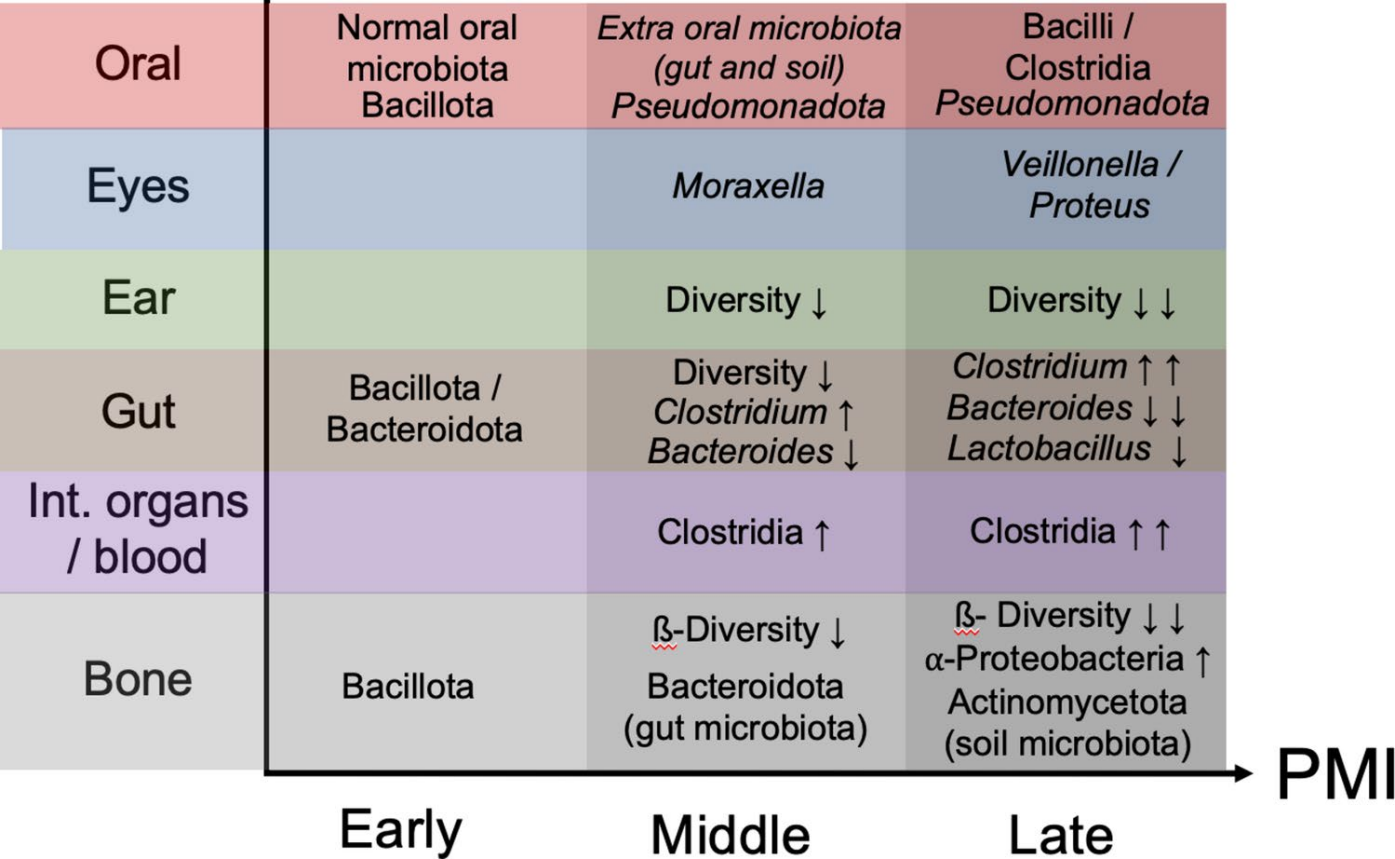
Summary of the changes in Bacteria over time in the mouth [21, 27], eyes [25], ears [24], gut [12] internal organs/blood [13, 14], and bone [22, 26]

This systematic review reveals that the microbiome from several body anatomical areas and the soil under the corpses have the potential to be used for PMI estimation. The microbial communities change dynamically and sequentially over time after death due to changes in environmental conditions surrounding the body and due to changes within the body, such as nutrient availability, oxygen, and humidity levels. Moreover, these microbiome changes vary according to the anatomical site. For example, *Clostridium*, *Bacteroides*, and *Lactobacillus* in the gut seem the most interesting, as their relative abundances change predictably with increasing PMI [12, 23] (Fig. 2). In the oral microbiome, however, regardless of the corpses’ initial different conditions, the same successional changes are observed as PMI increases, with a transition between oral microbiota, with a more abundance of bacteria from Bacillota phylum, to gut and soil microbiota, with a more abundance of bacteria from Pseudomonadota phylum [21, 27] (Fig. 2). As suggested by Adserias-Garriga et al. [21], the aerobic metabolism strongly determines bacterial colonization and, consequently, bacterial diversity during the fresh stage, and, therefore, oxygen consumption represents a significant driver for oral bacterial changes during decomposition. In both these habitats (oral and gut), the bloat state appears to mark a dramatic shift in the bacterial communities, maybe due to significant modifications in the environment and/or biological competition. Similar conclusions were reached when analyzing the corpse’s internal organs. Postmortem microbial proliferation is facilitated due to the nutrient-rich environment, and clostridia was appointed as a good predictor of PMI in internal organs and blood [13, 14] (Fig. 2). Interesting, Javan et al. [13] found distinct reproductive versus non-reproductive organ community profiles. The uterus and prostate were the last to decay during decomposition, whereas Lutz et al. [14] found bacterial genera similar among different organs within each sex but dissimilar between females and males (except in the oral cavity). These results may suggest that postmortem microbial succession should be explored to evaluate sex estimation. In the study by Zhang et al. [25], the highest accuracy in predicting the PMI was achieved when all five anatomic areas were used; therefore, microbial community analysis from different anatomical regions is expected to lead to better results.

The studies of Damann et al. [22] and Deel et al. [26] strongly suggest the potential of using bacterial community members as a temporal reference for estimating a PMI of skeletonized remains. Taxonomic succession occurs from partially skeletonized (mainly gut microbiota) to dry remains (mostly soil microbiota). Indeed, soil microbiome migrates progressively throughout decomposition, with a higher prevalence in skeletonized remains. This was also observed in samples from the hard palate, where *Streptophyta*, from Plantae kingdom, were common in 11 of 17 samples, suggesting a migration of the soil biota to the decomposing human remains [27]. On the other hand, as corpse decomposition advances, bacterial communities migrate from the cadavers to the soil, changing soil bacterial communities and microbial function immediately under the corpse for up to 2 years [7, 20]. Adserias-Garriga et al. [20] point out Bacillota as a phylum to explore in the soil immediately under human remains to estimate PMI during Tennessee summer conditions. Indeed, environmental factors are essential when estimating PMI, as the decomposer microbiome is affected by temperature, humidity, and other environmental factors. Therefore, in future studies, microbial succession during corpse decomposition should be evaluated in different seasons, geographic regions, and locations (inside, outside, underwater, etc.). Moreover, because the cadaver microbiome contaminates the soil microbiome under the corpse, the analysis of the soil can also have the potential to give a clue about corpse relocation.

Some studies explore microbial communities besides bacteria, namely eukaryotic organisms. In this case, results indicate that the prokaryotic analysis was more accurate in estimating PMI than the eukaryotic analysis. Also, most studies explored the microbial ecosystem using high-throughput sequencing, not a culture approach. In these cases, it was explored which target taxa could better estimate PMI. However, it was generally revealed that the community analysis is more informative than individual taxa. Bacterial diversity was a good predictor of PMI since it decreased during decomposition. This was particularly noticed in ear, gut, and bone analysis.

Machine-learning approaches were used in two studies, revealing them to be valuable tools for building predictive models. However, in some studies, the influence of environmental conditions and the year season was not evaluated, and the postmortem storage conditions of the cadavers did not represent the actual forensic conditions. For now, it is known that the decomposer microbiome differs between seasons (summer and spring) and is affected by cumulative precipitation. Much is still to explore, namely microbial changes related to burial time, climatic factors (such as direct sunlight), and underwater decomposition, among others. Similarly, knowing that humans have significant inter-individual microbiome differences (affected by diet and the antemortem environment, among other factors), it is still necessary to investigate whether these differences can affect PMI interpretation.

Moreover, most studies address decomposition stages, which can encompass several days (or months), making it difficult to estimate the PMI in a narrow interval. Future challenges should address this and describe changes using ADD or, even better, CHD. It is essential also to note the small sample size of most studies, limiting the interpretation of results and the drawing of conclusions.

## Conclusion

Postmortem microbial communities represent a potential forensic tool for estimating the PMI. As decomposition progresses, a dynamic temporal and spatial change occurs in the microbial community (in the corpse or the underneath soil), namely in the diversity and relative abundance of some taxa. Although there is still much to be done in this field, such as a multi-site collection of samples from the corpse, prospective studies with larger sample sizes and in different environmental conditions, comparing microbiome in the soil a short distance from the corpse to directly under the corpse, the results of the analyzed studies in this systematic review indicated that the microorganisms present in the cadaveric island succeed predictably over time, with markers between the stages of decomposition, constituting an innovative tool for PMI estimation.

In the future, it would be profitable to develop a protocol to guide the collection of microbial samples for PMI analyses. Differences between sex should also be addressed, as bacterial genera are similar among different organs within each sex but dissimilar between females and males (except in the oral cavity). These results may suggest that postmortem microbial succession should be explored to evaluate sex estimation.

We advise future studies to have a bacterial community approach to evaluate multiple anatomical areas and the soil immediately under the corpse. Also, it would be essential to assess the impact of the inter-individual microbiome differences in antemortem and the decomposition process under different environmental conditions. The data analysis for PMI estimation would benefit if machine learning approaches were used.

## Key points

1. Postmortem interval estimation can be very important in forensic scenarios.
2. Traditional methods may offer very broad estimations.
3. Human microbiome can be used for postmortem interval estimation.
4. Microbes are present in all habitats, including the most extreme ones.
5. Microbial communities respond predictably to environmental changes.

### Supplementary Information

Below is the link to the electronic supplementary material.

Supplementary file1 (DOCX 46.8 KB)
