# Supplementary material for: Microbiology and postmortem interval: a systematic review
Source: Forensic Sci Med Pathol. 2023 Oct 16;20(2):696–715. doi: 10.1007/s12024-023-00733-z (PMC11297127; doi:10.1007/s12024-023-00733-z)
Supplement: Supplementary file 1 — Supplementary file1 (DOCX 46.8 KB) [file 12024_2023_733_MOESM1_ESM.docx]

**Microbiology and postmortem interval: a systematic review**

|  | 1. Were the criteria for inclusion in the sample clearly defined? | 2. Were the study subjects and the setting described in detail? | 3. Was the exposure measured in a valid and reliable way? | 4. Were objective, standard criteria used for measurement of the condition? | 5. Were confounding factors identified? | 6. Were strategies to deal with confounding factors stated? | 7. Were the outcomes measured in a valid and reliable way? | 8. Was appropriate statistical analysis used? | Overall appraisal |
| --- | --- | --- | --- | --- | --- | --- | --- | --- | --- |
| (Adserias-Garriga, Hernandez, et al., 2017) | Yes | Yes | Yes | Yes | No | Not applicable | Yes | Yes | Include |
| (Adserias-Garriga, Quijada, et al., 2017) | Yes | Yes | Yes | Yes | No | Not applicable | Yes | Yes | Include |
| (Ashe et al., 2021) | Yes | Yes | Yes | Yes | Yes | No | Yes | Yes | Include |
| (Ceciliason et al., 2021) | Yes | Yes | Yes | Yes | Yes | Yes | Yes | Yes | Include |

**Supplementary** **Table 1:** Risk of bias analysis.

| (Damann et al., 2015) | Yes | Yes | Yes | Yes | No | Not applicable | Yes | Yes | Include |
| --- | --- | --- | --- | --- | --- | --- | --- | --- | --- |
| (DeBruyn & Hauther, 2017) | Yes | Yes | Yes | Yes | No | Not applicable | Yes | Yes | Include |
| (Deel et al., 2021) | Yes | Yes | Yes | Yes | Yes | No | Yes | Yes | Include |
| (Di Piazza et al., 2018) | Yes | Yes | Yes | Yes | Yes | Yes | Yes | Yes | Include |
| (Hauther et al., 2015) | Yes | Yes | Yes | Yes | No | Not applicable | Yes | Yes | Include |
| (Javan et al., 2016) | Yes | Yes | Yes | Yes | Yes | Yes | Yes | Yes | Include |
| (Johnson et al., 2016) | Yes | Yes | Yes | Yes | No | Not applicable | Yes | Yes | Include |
| (Lutz et al., 2020) | Yes | Yes | Yes | Yes | No | Not applicable | Yes | Yes | Include |
| (Singh et al., 2017) | Yes | Yes | Yes | Yes | No | Not applicable | Yes | Yes | Include |
| (Zhang et al., 2019) | Yes | Yes | Yes | Yes | No | Not applicable | Yes | Yes | Include |

Note: Articles were classified as to whether the risk of bias is "no", "yes" or "unclear" for each question present in the protocol, in all included articles. For each yes, a point was given, and articles scoring six or over were included in this review.

**References**

Adserias-Garriga, J., Hernandez, M., Quijada, N. M., Rodriguez Lazaro, D., Steadman, D., & Garcia-Gil, J. (2017). Daily thanatomicrobiome changes in soil as an approach of postmortem interval estimation: An ecological perspective. *Forensic Sci Int*, *278*, 388-395. <https://doi.org/10.1016/j.forsciint.2017.07.017>

Adserias-Garriga, J., Quijada, N. M., Hernandez, M., Rodriguez Lazaro, D., Steadman, D., & Garcia-Gil, L. J. (2017). Dynamics of the oral microbiota as a tool to estimate time since death. *Mol Oral Microbiol*, *32*(6), 511-516. <https://doi.org/10.1111/omi.12191>

Ashe, E. C., Comeau, A. M., Zejdlik, K., & O'Connell, S. P. (2021). Characterization of Bacterial Community Dynamics of the Human Mouth Throughout Decomposition via Metagenomic, Metatranscriptomic, and Culturing Techniques. *Front Microbiol*, *12*, 689493. <https://doi.org/10.3389/fmicb.2021.689493>

Ceciliason, A. S., Andersson, M. G., Lundin, E., & Sandler, H. (2021). Microbial neoformation of volatiles: implications for the estimation of post-mortem interval in decomposed human remains in an indoor setting. *Int J Legal Med*, *135*(1), 223-233. <https://doi.org/10.1007/s00414-020-02436-4>

Damann, F. E., Williams, D. E., & Layton, A. C. (2015). Potential Use of Bacterial Community Succession in Decaying Human Bone for Estimating Postmortem Interval. *J Forensic Sci*, *60*(4), 844-850. <https://doi.org/10.1111/1556-4029.12744>

DeBruyn, J. M., & Hauther, K. A. (2017). Postmortem succession of gut microbial communities in deceased human subjects. *PeerJ*, *5*, e3437. <https://doi.org/10.7717/peerj.3437>

Deel, H., Emmons, A. L., Kiely, J., Damann, F. E., Carter, D. O., Lynne, A., Knight, R., Xu, Z. Z., Bucheli, S., & Metcalf, J. L. (2021). A Pilot Study of Microbial Succession in Human Rib Skeletal Remains during Terrestrial Decomposition. *mSphere*, *6*(4), e0045521. <https://doi.org/10.1128/mSphere.00455-21>

Di Piazza, S., Zotti, M., Barranco, R., Cecchi, G., Greco, G., & Ventura, F. (2018). Post-mortem fungal colonization pattern during 6 weeks: Two case studies. *Forensic Sci Int*, *289*, e18-e23. <https://doi.org/10.1016/j.forsciint.2018.05.037>

Hauther, K. A., Cobaugh, K. L., Jantz, L. M., Sparer, T. E., & DeBruyn, J. M. (2015). Estimating Time Since Death from Postmortem Human Gut Microbial Communities. *J Forensic Sci*, *60*(5), 1234-1240. <https://doi.org/10.1111/1556-4029.12828>

Javan, G. T., Finley, S. J., Can, I., Wilkinson, J. E., Hanson, J. D., & Tarone, A. M. (2016). Human Thanatomicrobiome Succession and Time Since Death. *Sci Rep*, *6*, 29598. <https://doi.org/10.1038/srep29598>

Johnson, H. R., Trinidad, D. D., Guzman, S., Khan, Z., Parziale, J. V., DeBruyn, J. M., & Lents, N. H. (2016). A Machine Learning Approach for Using the Postmortem Skin Microbiome to Estimate the Postmortem Interval. *PLoS One*, *11*(12), e0167370. <https://doi.org/10.1371/journal.pone.0167370>

Lutz, H., Vangelatos, A., Gottel, N., Osculati, A., Visona, S., Finley, S. J., Gilbert, J. A., & Javan, G. T. (2020). Effects of Extended Postmortem Interval on Microbial Communities in Organs of the Human Cadaver. *Front Microbiol*, *11*, 569630. <https://doi.org/10.3389/fmicb.2020.569630>

Singh, B., Minick, K. J., Strickland, M. S., Wickings, K. G., Crippen, T. L., Tarone, A. M., Benbow, M. E., Sufrin, N., Tomberlin, J. K., & Pechal, J. L. (2017). Temporal and Spatial Impact of Human Cadaver Decomposition on Soil Bacterial and Arthropod Community Structure and Function. *Front Microbiol*, *8*, 2616. <https://doi.org/10.3389/fmicb.2017.02616>

Zhang, Y., Pechal, J. L., Schmidt, C. J., Jordan, H. R., Wang, W. W., Benbow, M. E., Sze, S. H., & Tarone, A. M. (2019). Machine learning performance in a microbial molecular autopsy context: A cross-sectional postmortem human population study. *PLoS One*, *14*(4), e0213829. <https://doi.org/10.1371/journal.pone.0213829>
